# Transcriptome analysis during vernalization in wheat (*Triticum aestivum* L.)

**DOI:** 10.1186/s12863-023-01144-3

**Published:** 2023-08-10

**Authors:** Jiao Wang, Lei Sun, Hongwei Zhang, Bo Jiao, Haibo Wang, Shuo Zhou

**Affiliations:** Institute of Biotechnology and Food Science, Hebei Academy of Agriculture and Forestry Sciences/Plant Genetic Engineering Center of Hebei Province, Shijiazhuang, China

**Keywords:** Wheat (*Triticum aestivum* L.), Vernalization, Shiluan02-1, Transcriptome analysis, RNA-seq

## Abstract

**Background:**

Vernalization, as a vital process in the life cycle of winter cereal, has important effects on floral organ formation and flowering time. Many morphological changes together with molecular changes occur during the vernalization period. Here, we used transcriptome sequencing to analyze the transcriptomic changes in wheat leaves before, during and after vernalization using the winter wheat cultivar ‘Shiluan02-1’.

**Results:**

A total of 16,370 differentially expressed genes were obtained across different vernalization periods. Gene Ontology enrichment analysis revealed that photoperiodism, photoprotection, photosynthesis, lipid transport and biosynthetic process, and chlorophyll metabolic process were closely related to vernalization. In addition, *AP2/ERF*, *C2H2*, *bHLH*, *WRKY*, *MYB*, *MYB-related*, and *NAC* transcription factors were significantly enriched during vernalization, and the transcription factor expression patterns suggested the intricate regulation of transcription factor modules in plant vernalization pathways. Analysis of gene expression patterns of the MADS-box transcription factor genes showed different expression patterns during vernalization phases, among which *VERNALIZATION1* (*VRN1*) genes were found to gradually increase during vernalization periods from V0 to V35, while decline in the V42 phase, then increase after vernalization. The *Tavrt-2* gene cooperated with *Tavrn1* to regulate flowering induced by vernalization, and its expression level was rapidly increased by vernalization but declined in the V42 phase and then increased after vernalization. Some genes from the *ICE*-*CBF*-*COR* pathway were also identified, and additional analysis indicated that some key genes related to phytohormone biosynthesis and signal transduction were enriched during the vernalization period, such as gibberellic acid, ethylene, abscisic acid and jasmonic acid biosynthesis and signaling pathway genes.

**Conclusions:**

Our study provides valuable molecular information for future studies on wheat vernalization regulation and also serves as an excellent reference for future wheat breeding.

**Supplementary Information:**

The online version contains supplementary material available at 10.1186/s12863-023-01144-3.

## Background

Plants begin reproductive growth after a period of vegetative growth, which is regulated by various exogenous and endogenous signals that ultimately induce flowering. In this process, a regulatory network ensures plants can successfully progress from vegetative to reproductive growth. Five pathways that control flowering have been identified, namely the autonomous [1], photoperiod [2], vernalization [3], gibberellin (GA) [4], and age pathways [5].

For winter cereals, low temperature is an inevitable factor that directly affects plant development and flowering together with long days, which ultimately contributes greatly to seed production. Genes controlling flowering time via the vernalization pathway have been identified in the model plant *Arabidopsis thaliana* and in the overwintering crop *Triticum aestivum* L*.* [6, 7]*.* In *A. thaliana*, *FLOWERING LOCUS C* (*FLC*), a MADS*-*box transcription factor (TF), regulates the vernalization pathway via *FLC* mRNA level, and FRIGIDA represses flowering by activating *FLC* to increase *FLC* mRNA abundance [8–10]. In wheat, the vernalization pathway is mainly regulated by three genes: *VERNALIZATION1* (*VRN1*), *VERNALIZATION2* (*VRN2*), and *VERNALIZATION3* (*VRN3*). *VRN1* is a flowering activator and has three homologs [11], *VRN2* acts as a flowering repressor and is down-regulated by vernalization, *VRN3* has been verified to be *Flowering Locus T* (*FT*), which encodes a mobile signal protein moving from the leaf to the apical meristem to induce flowering [12]. During vernalization, *VRN1* expression increases to repress *VRN2* expression, which in turn releases FT during long days, thus inducing flowering [13].

A previous study identified vernalization-responsive genes via transcriptome analysis using the winter wheat cultivar ‘Jing841’ and a spring wheat cultivar ‘Liaochun10’, and 636 differentially expressed genes (DEGs) were identified, including *VRN-1*, *COR14a, IRIP*, *unigene1806*, and *Cl18953* [14]. A transcriptome analysis of CBW0101 spring durum wheat under cold (5 °C) treatment at the reproductive stage identified DEGs involved in many processes, including photosynthetic activity, lipid and carbohydrate synthesis, accumulation of amino acids and seed proteins, and TFs and long non-coding RNAs (lncRNAs) induced by cold stress [15]. A transcriptome analysis of *Dongnongdongmai1* (*Dn1*) comprehensively identified the key genes involved in cold signal transduction and carbohydrate metabolism in *Dn1* under cold stress [16]. A transcriptional analysis also found that *VRN-A1* genetically promoted floral development, and the gradual up-regulation of abscisic acid-dependent and C-REPEAT-BINDING FACTOR pathways regulated delayed shoot apex development and the induction of cold tolerance [17]. Another transcriptome analysis in winter wheat identified some up-regulated DEGs, including *CBFIIId-12.1*, WRKY transcription factor 55-like, and genes related to the jasmonate signaling pathway using the Lithuanian winter wheat cultivar ‘Gaja DS’ [18].

Here, we explored the transcriptomes of wheat leaves using RNA sequencing (RNA-seq) before, during and after vernalization in the cultivar ‘Shiluan02-1’. The DEG analysis identified characteristic genes with different expression patterns and TFs that have been reported to be involved in vernalization-controlled flowering. The expression patterns of phytohormone-related genes showed that they participated synergistically in response to vernalization. Our study provides a new reference for flowering regulation via the vernalization pathway in wheat and also provides a foundation for future studies on the genetic regulation of flowering time and yield in wheat.

## Materials and methods

### Plant material preparation

The winter wheat cultivar ‘Shiluan02-1’, cultivated by Prof. Zhanjing Huang (Hebei Normal University) and kept in our laboratory, was used in this study. The seeds were disinfected in 70% (V/V) ethanol for 1 min and 2% (V/V) NaClO for 10 min, and then washed in sterile water three times, following which they were germinated in a petri dish for 24 h in the dark. The germinated seeds were sown in a matrix and grown in an illumination incubator at 22 °C under long-light conditions (LL, 16 h/8 h) for 2 weeks. Two newly developed leaves were collected and named V0, and the remaining seedlings were transferred into the 4 °C illumination incubator for vernalization under LL conditions for 6 weeks. Then two newly developed leaves were collected every week during the vernalization period and named V7, V14, V21, V28, V35 and V42, which represented vernalization for 1, 2, 3, 4, 5, and 6 weeks, respectively. Three biological replicates were collected for each vernalization period. The remaining seedlings were left to grow in the illumination incubator at 22 °C under LL conditions for 1 week until the end of sampling at V42N7. The collected samples were quickly frozen in liquid nitrogen and stored at − 80 °C until use.

### Library preparation for transcriptome sequencing

The library was constructed using the NEBNext® Ultra™ RNA Library Prep Kit (NEB, USA), with a slight modification in the process. VAHTS mRNA Capture beans (Vazyme Biotech, Nanjing China) were used for the isolation of mRNA, and VAHTS DNA clean beans (Vazyme Biotech, Nanjing, China) were used for the purification of double-stranded cDNA and PCR products, and the selection of fragments of connecting products. The constructed cDNA library of wheat leaves subjected to different vernalization treatments was then sequenced using the Illumina HiSeq 4000 sequencing system (Illumina, USA).

### Quality control and mapping of reads

Raw reads were filtered by adopting FASTPv0.23 [19] with default parameters to remove adapters and low-quality reads to generate clean reads. Clean reads were mapped to the reference genome of *Triticum aestivum* cv. Chinese Spring (IWGSC RefSeq v2.1) with default parameters. An index for the reference genome was constructed using Hisat2 v2.2.1 [20], with the parameters set to default. The double ends of the clean reads were compared to the reference genome. The bam files were compressed using Samtools v1.10 [21] and sorted to build the index. The sequencing results were evaluated with Qualimap v2.2.1 [22], and FeatureCounts v1.5.0 [23] was used to calculate the number of clean reads mapped to each gene and standardize the expression level with transcripts per kilobase per million mapped reads (TPM). Genes with low expression were then filtered using TPM > 1 for expression quantification.

### Correlation and principal component analysis

Correlation analysis was performed using Spearman’s correlation coefficient (SCC) based on the R function ‘cor’ within and between the samples. The principal component analysis (PCA) used the R package PCA tools v2.8.0 [24].

### Identification and functional annotation of DEGs

The DEGs between the materials were identified using the R package DEseq2 v1.20.0. The adjusted *P*-value (padj) < 0.05 and the Benjamini–Hochberg method were used together with |log2fold change|> 1 to screen the significant DEGs. The R package VennDiagram [25] was used to screen significant DEGs that were common among all samples and that were specific to each sample.

### Gene Ontology (GO) enrichment and Kyoto Encyclopedia of Genes and Genomes (KEGG) analyses

The GO analysis was conducted using the local functional annotations database (http://eggnog6.embl.de/download/emapperdb-5.0.2/) by eggNOG-Mapper V2 [26]**.** Functional annotations were then packaged in the R package OrgDb based on the R package Annotation Forge [27]. The K numbers of all wheat proteins were obtained by KofamKOALA [28] using the exec_annotation command embedded in kofamscan based on the local KOfam database (ftp://ftp.genome.jp/pub/db/kofam/). The KEGG analysis was performed by establishing the link between K numbers and KO pathways in wheat (https://www.genome.jp/kegg-bin/get_htext?taes00001). The GO and KEGG enrichment analyses between different materials used the R package clusterProfiler [29] with padj < 0.05 as the threshold.

### Time series clustering

To compare time-series gene expression data, all of the 16,370 DEGs between any two time points were gathered and unsupervised clustered by the fuzzy c-means algorithm implemented in the R package Mfuzz [30]**.**

### Genome-wide identification of the TF family and identification of genes with certain domains

The TFs were predicted with iTAK [31] and clustered into 49 families using the PlantTFDB [32] databases based on their protein sequences. The over-representation analysis of TF genes in each of the 10 clusters considered *P*-values < 0.05 as significant. Proteins containing both MADS domains (PF00319) and K domains (PF01486), of which HMM was derived from Pfam (the protein family database, MSA), were filtered by HMMER. After removing repetitive sequences, the remaining sequences were predicted to contain both domains. The expression patterns of some of the filtered genes during vernalization were visualized by GraphPad Prism 7. *ICE*-*CBF*-*COR* genes were previously predicted. The Entrez IDs of the candidate genes from IWGSCV1.1 were converted by blast and confirmed based on their chromosome positions. Genes with low expression were filtered based on the sum of TPM > 1, and the expression patterns were visualized in a heatmap.

## Results

### Transcriptome analysis of wheat leaves during the vernalization phase

To investigate the transcriptomic changes during the vernalization phase in winter wheat, we generated a set of comprehensive transcriptomes. Three biological replicates at each sampling point, which included 24 samples of the winter wheat cultivar ‘Shiluan02-1’, were collected for RNA-seq assays. A total of 1,880,999,462 high-quality (Q30 > 95%) clean reads were obtained, with an average of 78,374,977 reads (> 8 Gb) for biological replicates. These reads were mapped to the reference genome of CS (IWGSC RefSeq v2.1), with an average rate of 82.9% unique alignment (Table 1). The gene expression level was calculated using the Transcripts Per Kilobase Million uniquely mapped reads (TPM).

**Table 1:** Sequencing data statistics of all processed samples

Sample	Total reads	Clean reads	Q20(%)	Q30(%)	Total mapped (%)	Uniquely mapped (%)
V0-1	69,355,552	68,486,966	98.6112	95.701	96.31	79.7
V0-2	68,988,248	68,332,394	98.5801	95.647	96.31	79.29
V0-3	65,984,022	65,240,054	98.6151	95.6866	95.72	80.42
V7-1	73,475,276	72,547,046	98.5752	95.6077	96.19	79.92
V7-2	67,645,124	66,972,748	98.4983	95.3818	96.41	83.79
V7-3	72,495,982	71,372,916	98.357	95.0803	96.17	79.7
V14-1	86,970,206	86,331,254	98.6127	95.7145	96.51	82.16
V14-2	86,797,034	86,077,194	98.592	95.597	96.39	83.28
V14-3	72,457,096	71,787,602	98.6468	95.8128	96.19	83.89
V21-1	66,934,652	65,984,566	98.4537	95.1008	94.79	79.47
V21-2	75,803,704	74,920,284	98.5827	95.3695	96.46	83.34
V21-3	75,765,136	74,995,838	98.6301	95.4975	96.6	79.75
V28-1	106,245,964	104,942,400	98.5931	95.7309	95.95	83.64
V28-2	102,413,134	101,192,968	98.5488	95.6137	95.8	83.88
V28-3	125,349,336	123,987,394	98.6655	95.9047	96.08	83.29
V35-1	86,094,522	85,521,146	98.7371	95.7745	96.27	84.45
V35-2	76,789,204	76,196,982	98.7434	95.7903	96.26	85.13
V35-3	66,757,970	66,069,598	98.6951	95.6759	96.29	85.88
V42-1	70,479,700	69,583,288	98.5746	95.3295	95.75	84.63
V42-2	76,122,046	75,134,352	98.6207	95.4866	95.81	85.73
V42-3	76,717,152	75,947,390	98.5986	95.432	95.88	85.14
V42N7-1	71,034,372	70,538,296	98.6602	95.5688	96.23	85.47
V42N7-2	81,253,582	80,661,102	98.5225	95.1843	96.14	85.29
V42N7-3 Total	79,160,162 1,901,089,176	78,175,684 1,880,999,462	98.6155	95.4432	96.28	83.47

**Fig. 1:**
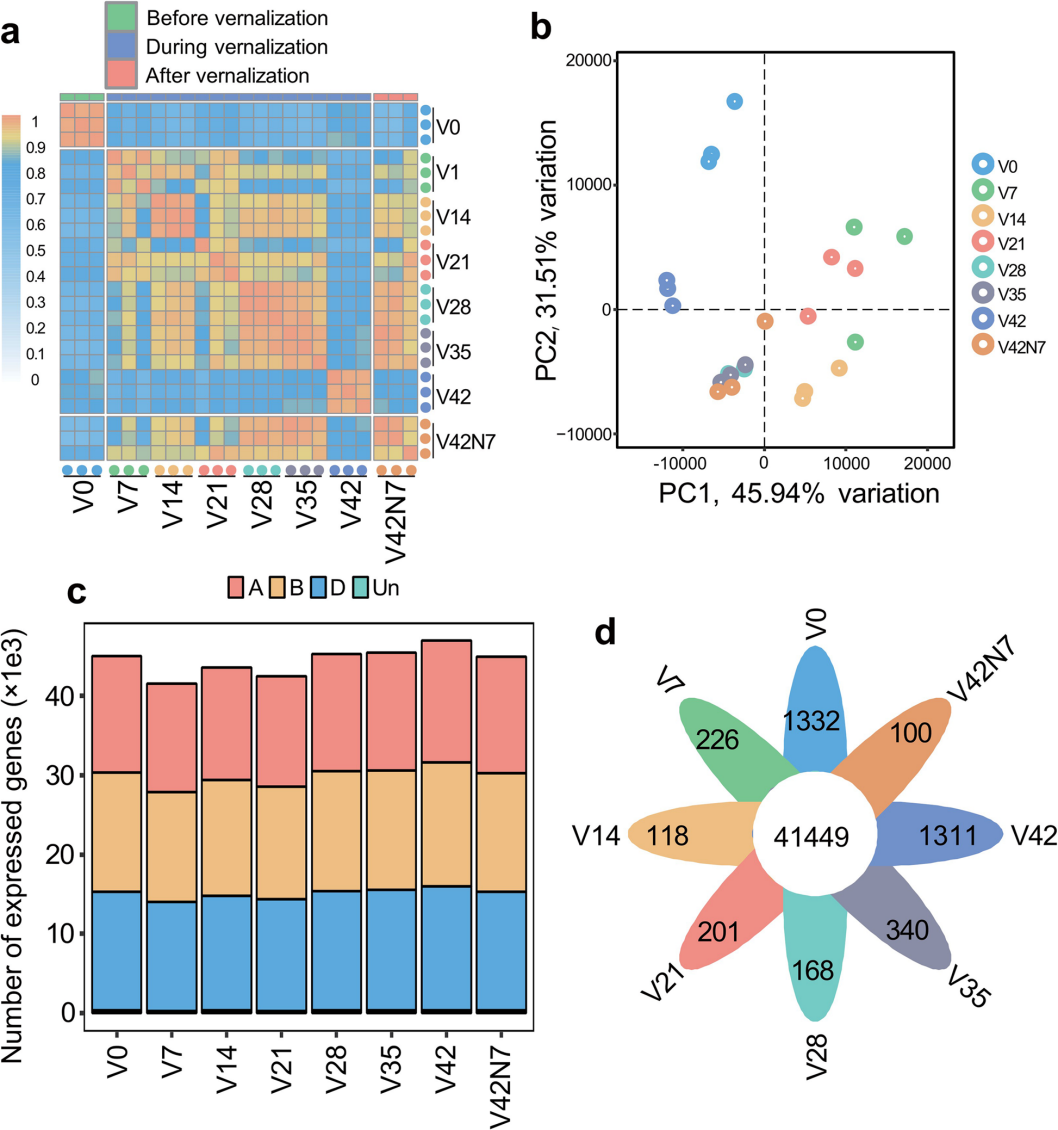
Correlation analysis and principal component analysis (PCA) of all transcripts and expressed genes in the genome. **a** Correlation analysis of expressed genes among three biological replicates of different samples from different treatments. The dot indicates one biological replicate, and the scale bar represents Spearman’s correlation coefficient. **b** PCA plot of all transcriptome results. The first and second PCs explained 45.94% and 31.51% variance, respectively. **c** Expressed gene numbers in the A, B, and D subgenomes of different samples from different treatments. **d** The flower plot displays all expressed gene numbers (in the center) among all samples and genes specifically expressed in the petals at different time points

A PCA was performed based on the TPM values of three biological replicates from different treatments. The TPM values of all 24 samples were presented in box plots. Samples from different treatments showed a higher expression correlation in transcriptomes and biological processes. The SCC and PCA analysis presented conserved patterns, and the transcriptomes of the samples clustered, especially samples V35 and V28 (Fig. 1a and b).

Transcriptome expression profiling of all the samples revealed the genes that were expressed in samples with different treatments. A total of 81,879 expressed genes (TPM > 1) were found during the vernalizing phase. The numbers of expressed genes were similar among the three sub-genomes (ABD). And the number of expressed genes decreased in V7, V14, V21, and V42N7, but increased in V42 when compared with V0 (Fig. 1c). Of these expressed genes, 41,449 genes were found to produce mRNAs in all samples, while only 3,796 genes were expressed in a specific phase, comprising 1,332 in the V0 phase, 226 in the V7 phase, 118 in the V14 phase, 201 in the V21 phase, 168 in the V28 phase, 340 in the V35 phase, 1,311 in the V42 phase, and 100 in the V42N7 phase (Fig. 1d).

V0, expressed before vernalization; V7, V14, V21, V28, V35 and V42, expressed after vernalization for 7, 14, 21, 28, 35, and 42 days, respectively; V42N7, normal growth for 7 days after vernalization for 42 days. -1, -2, and -3 indicate the three duplications of each sample.

### Identification of DEGs among adjacent materials

**Fig. 2:**
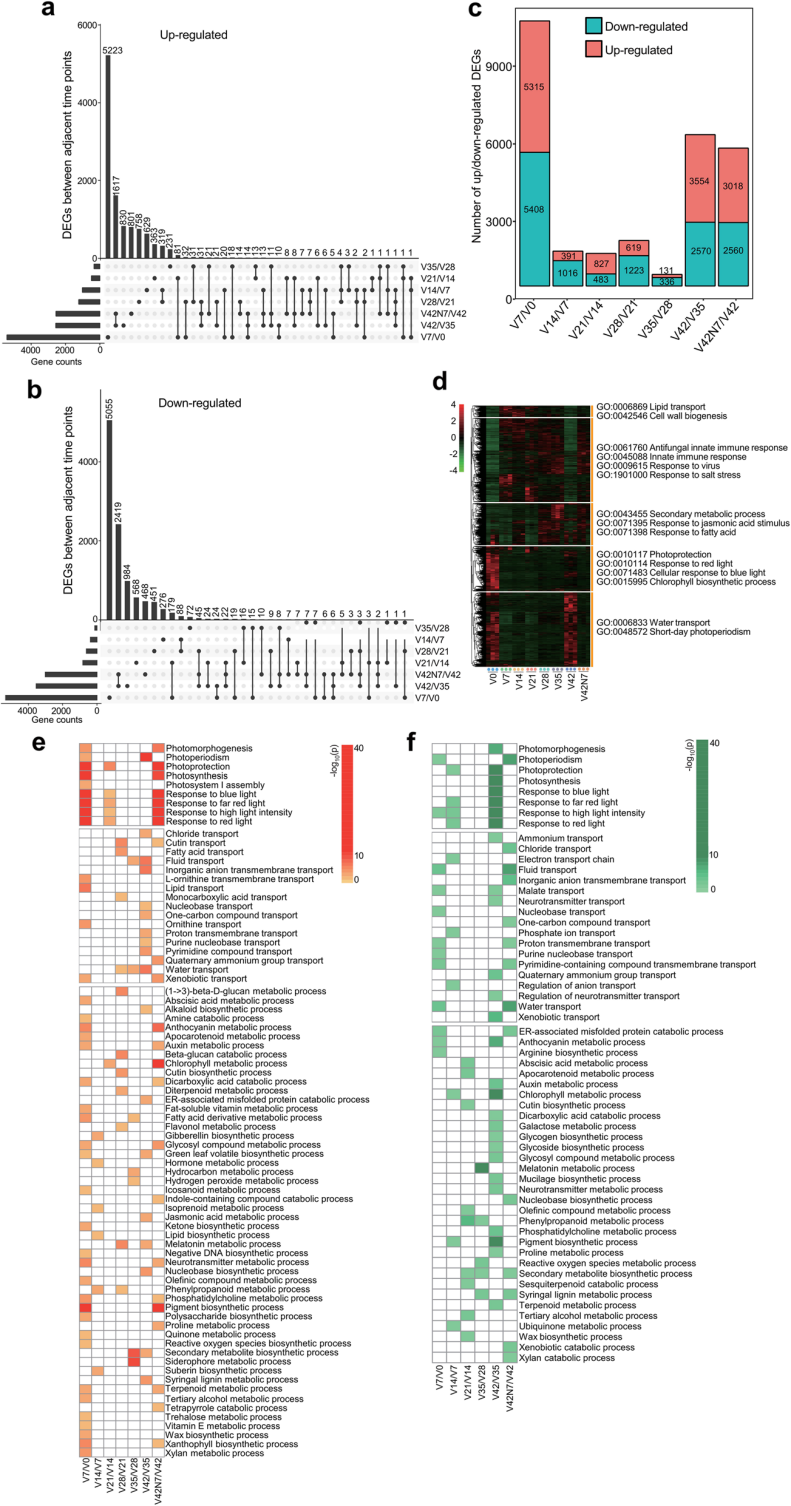
Differentially expressed gene (DEG) analysis between adjacent processed materials. **a** UpSet plot diagram of up-regulated DEGs expressed between adjacent time points, and the number of DEGs shared by different comparisons (V7 vs. V0; V14 vs. V7; V21 vs. V14; V28 vs. V21; V35 vs. V28; V42 vs. V35; and V42N7 vs. V42). **b** Down-regulated DEGs expressed between adjacent time points, and the number of DEGs shared by different comparisons. **c** The number of DEGs between adjacent time points. **d** Cluster dendrogram of all processed samples according to different expression patterns. **e** GO enrichment analysis of up-regulated DEGs between adjacent time points (V7 vs. V0; V14 vs. V7; V21 vs. V14; V28 vs. V21; V35 vs. V28; V42 vs. V35; and V42N7 vs. V42) according to different biological processes. **f** GO enrichment of down-regulated DEGs between adjacent time points (V7 vs. V0; V14 vs. V7; V21 vs. V14; V28 vs. V21; V35 vs. V28; V42 vs. V35; and V42N7 vs. V42) according to different biological processes

To investigate molecular differences during the vernalizing phase, seven pairwise comparisons were made between adjacent samples. A total of 16,370 genes were identified as significant DEGs between adjacent samples, comprising 10,723 in V7 vs. V0, 1,407 in V14 vs. V7, 1,310 in V21 vs. V14, 1,842 in V28 vs. V21, 467 in V35 vs. V28, 6,124 in V42 vs. V35, and 8,596 in V42N7 vs. V42 (Fig. 2c, Table S1). Moreover, the numbers of up/down-regulated genes were calculated, and V42N7 vs. V42 and V42 vs. V35 shared the most up-regulated (1,617) and down-regulated (2,419) DEGs (Fig. 2a and b), using an adjusted *P*-value < 0.05 and an absolute value of log^2^ fold change ≥ 1 as thresholds. The number of DEGs in V7 vs. V0 was remarkably higher than in the other pairwise comparisons, indicating that more complex physiological procedures occurred in the initial stage of vernalization. All 24 samples were clustered in five groups in a cluster dendrogram analysis according to different expression patterns (Fig. 2d).

To further explore the biological processes that these DEGs were involved in, GO enrichment annotations for up/down-regulated DEGs between adjacent time points were produced according to different biological processes (Table S2). The results indicated that the up-regulated and down-regulated DEGs in the biological process pathways of photoprotection, photosynthesis, response to blue light, response to far-red (FR) light, response to high light intensity, and response to red light were enriched in V7 vs. V0 and V42N7 vs. V42 for up-regulated DEGs and in V42 vs. V35 for down-regulated DEGs. Furthermore, genes related to the chlorophyll metabolic process, pigment biosynthetic process, and secondary metabolite biosynthetic process were also significantly enriched in up/down-regulated DEGs. Siderophore metabolic process was expressed abundantly in the group of up-regulated DEGs, and lipid transport and biosynthetic process were specifically enriched in V7 vs. V0 and V14 vs. V7 for up-regulated DEGs (Fig. 2e and f).

### The KEGG enrichment analysis of DEGs

**Fig. 3:**
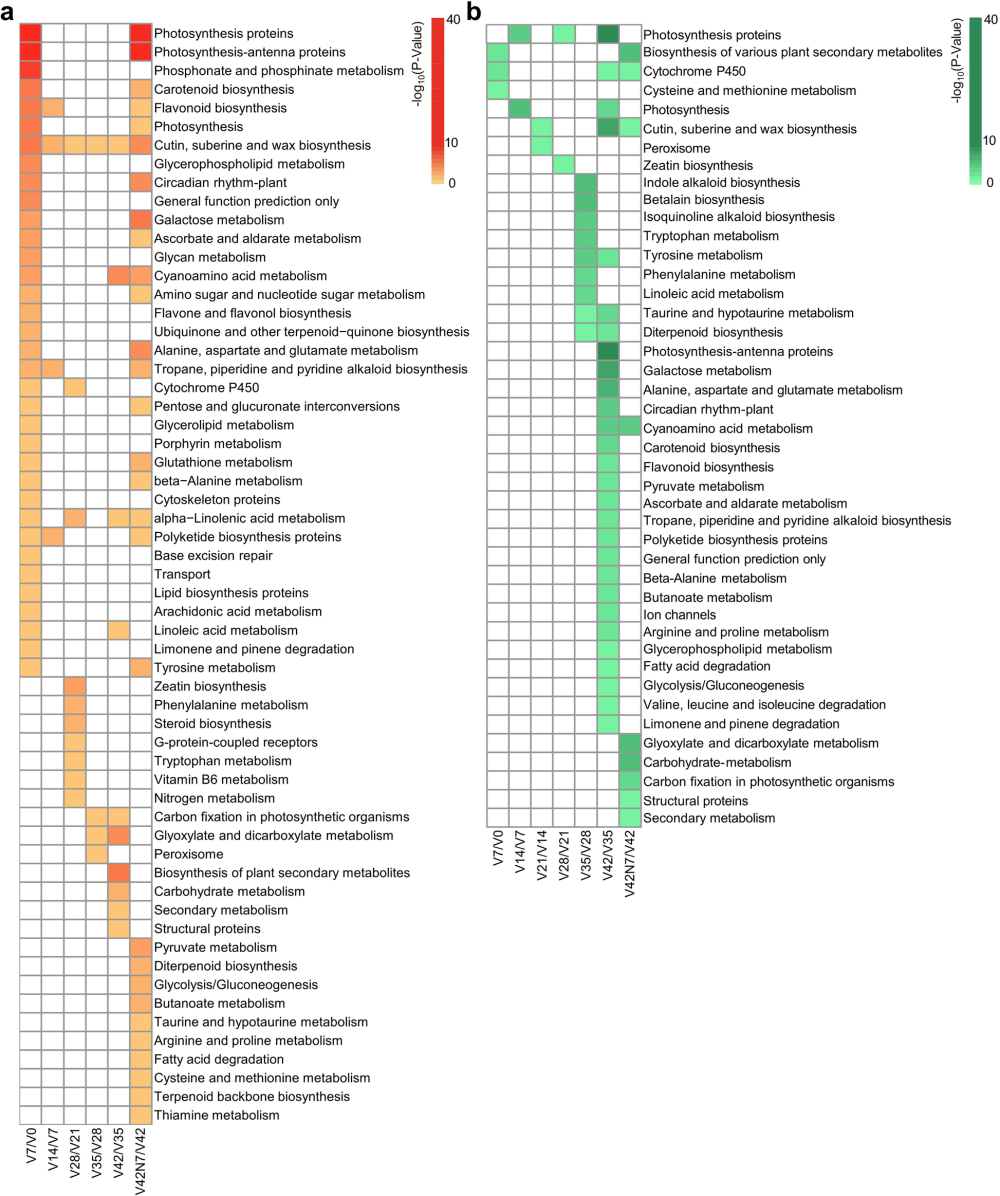
Kyoto Encyclopedia of Genes and Genomes (KEGG) analysis between adjacent samples. **a** KEGG analysis of up-regulated DEGs between adjacent samples. **b** KEGG analysis of down-regulated DEGs between adjacent samples. The gene expression level is based on the scaled TPM values

To better explain the molecular associations among the DEGs, we performed KEGG analyses between adjacent vernalization phases (V7 vs. V0, V14 vs. V7, V21 vs. V14, V28 vs. V21, V35 vs. V28, V42 vs. V35 and V42N7 vs. V42). A total of 59 and 43 different KEGG enrichment pathways were identified for up- and down-regulated DEGs, respectively (Tables S3 and S4). For up-regulated DEGs, 35, 4, 10, 4, 10, and 29 KEGG pathways were identified between V7 vs. V0, V14 vs. V7, V28 vs. V21, V35 vs. V28, V42 vs. V35, and V42N7 vs. V42, respectively, in which photosynthesis proteins and photosynthesis-antenna proteins pathways were enriched at vernalization for 1 week and after vernalization for 1 week (Fig. 3a). Regarding down-regulated DEGs, 3, 2, 2, 2, 9, 28, and 9 KEGG enrichment pathways were identified between V7 vs. V0, V14 vs. V7, V21 vs. V14, V28 vs. V21, V35 vs. V28, V42 vs. V35, and V42N7 vs. V42, respectively (Fig. 3b), and photosynthesis proteins were most enriched at vernalization for 2, 4, and 6 weeks. Photosynthesis-antenna proteins were only enriched at vernalization for 6 weeks. Photosynthesis and photosynthesis-antenna proteins pathways may be closely related to the vernalization pathway. There were also other significantly enriched KEGG pathways related to vernalization, such as phosphonate and phosphinate metabolism, glyoxylate and dicarboxylate metabolism, and biosynthesis of plant secondary metabolites.

### Expression clusters analysis

**Fig. 4:**
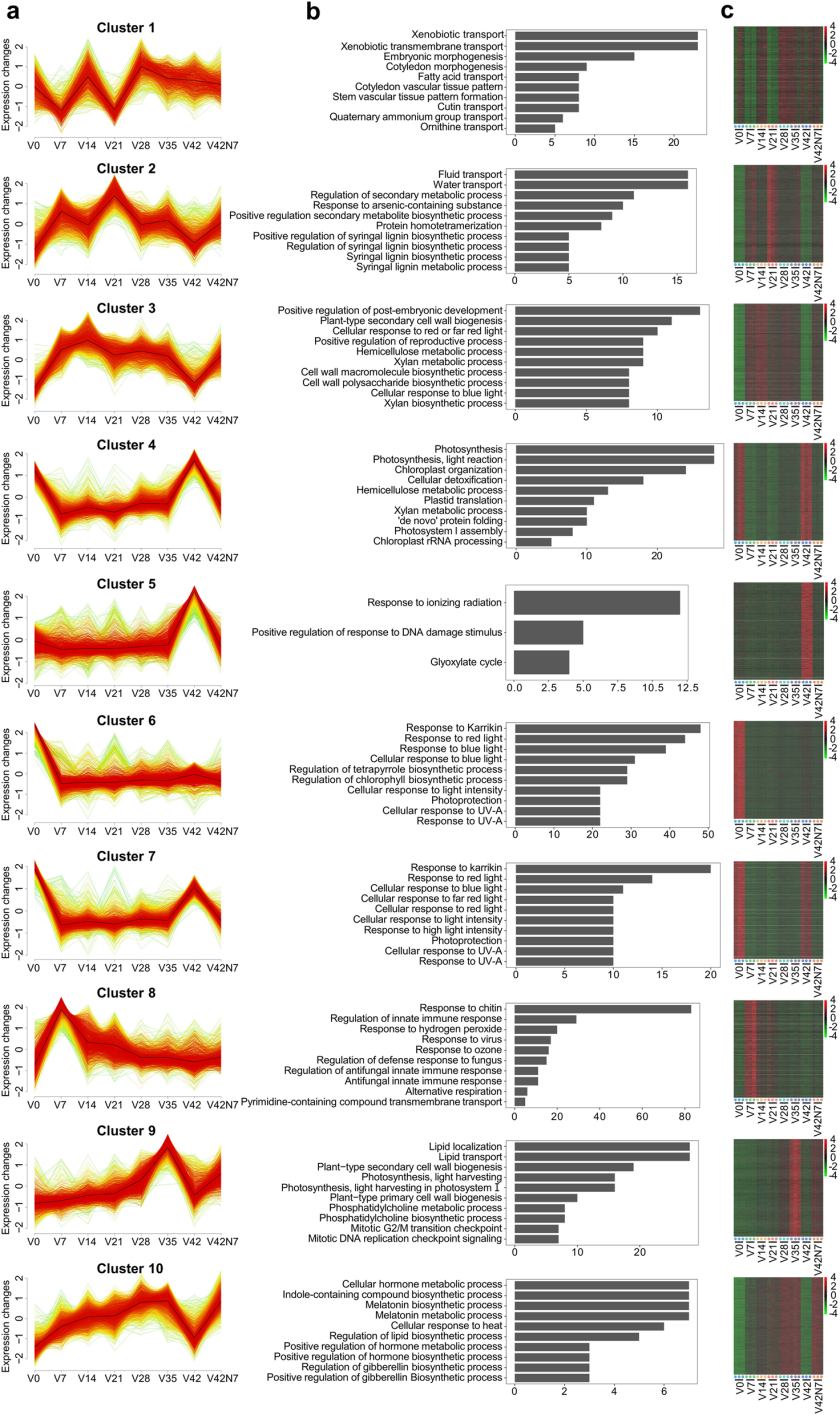
Co-expression clusters, GO, and heatmap analysis of genes in each of the 10 clusters. **a** The red curves show the expression trends of genes in 10 clusters (C1–C10) during the eight time points. The dark curve represents the average expression level of genes in each cluster. The *x*-axis indicates different samples from different treatments, and the *y*-axis indicates the expression change level. **b** The most enriched biological process GO terms in 10 clusters. **c** Heatmaps illustrating the differential expression patterns of genes in 10 clusters of all processed samples

To identify the major dynamics associated with vernalization, time-series trend analysis was performed to group genes according to similar expression profiles. Genes were divided into 10 clusters (C1-C10) with different expression patterns, and 1394, 1585, 1217, 1182, 3165, 1621, 1321, 2596, 1285, and 1004 genes were contained in the C1 to C10 clusters, respectively. Cluster 1 comprised genes that were down-regulated at the beginning of vernalization and that exhibited the lowest expression level in V7. These genes showed two low peaks in V7 and V21, were highly expressed in V28, and then maintained the same expression levels during the vernalization period from V28 to V42N7. Furthermore, these genes were most enriched in the process of xenobiotic transport, fatty acid transport, cutin transport, quaternary ammonium group and ornithine transport, cotyledon and embryonic morphogenesis, and cotyledon and stem vascular tissue pattern formation. For Cluster 2, a large number of genes were enriched in the fluid transport, water transport, regulation of the secondary metabolic process, response to an arsenic-containing substance, protein homo-tetramerization, and syringa L lignin biosynthetic/metabolic process and were rapidly up-regulated and showed four high expression peaks in V7, V21, V35, and V42N7. Genes in Cluster 3 were rapidly up-regulated to the highest expression level in V14 but were down-regulated in V42 and then up-regulated after vernalization. Furthermore, these were linked to the processes of positive regulation of post-embryonic development, plant-type secondary cell wall biogenesis, cellular response to red or far-red and blue light, positive regulation of reproductive, hemicellulose metabolic, xylan biosynthetic and metabolic process, cell wall macromolecule, and polysaccharide biosynthetic process. Genes in Cluster 4 were rapidly down-regulated by vernalization to the lowest expression level in V7 and maintained their expression level during the vernalization period from V7 to V35, following which they were up-regulated in V42 but declined after vernalization. These genes were involved in the processes of photosynthesis, light reaction, chloroplast organization, cellular detoxification, hemicellulose metabolic process, plastid translation, xylan metabolic process, ‘de novo’ protein folding, photosystem I assembly, and chloroplast rRNA processing. Cluster 5 showed one peak in the V42 phase, and the genes were enriched in response to ionizing radiation, positive regulation of response to DNA damage stimulus, and glyoxylate cycle. Cluster 6 genes were rapidly down-regulated by vernalization and exhibited the lowest expression level in V7 and maintained this expression level during the vernalization period from V7 to V42N7. The genes in this cluster encode proteins with similar functions in response to Karrikin and response to light processes, such as response to red light, blue light, UV − A, light intensity, photoprotection processes, and regulation of tetrapyrrole chlorophyll biosynthetic process. Cluster 7 showed similar expression patterns with Cluster 4, and the genes in this cluster were most enriched in response to Karrikin and response to the light process, such as blue light, red light and FR light, UV − A, light intensity, and photoprotection processes. When plants experience low temperatures, the immune response will be initiated immediately, and so genes in Cluster 8 were mainly related to immune response processes, such as response to chitin, regulation of innate immune response, response to hydrogen peroxide, response to virus and ozone, regulation of defense response to fungus, and antifungal innate immune response. These genes were rapidly induced in the V7 phase but declined in expression in V14 and then maintained their expression level thereafter. Cluster 9 had one peak in V35, and the genes in this cluster were most enriched in lipid localization and transport, plant-type primary and secondary cell wall biogenesis, photosynthesis, light harvesting, phosphatidylcholine biosynthetic and metabolic process, mitotic G2/M transition checkpoint, and mitotic DNA replication checkpoint signaling, which were gradually induced by vernalization to the highest expression level in V35, but declined in V42 and then increased after vernalization in V42N7. Cluster 10 showed two low peaks in the V0 and V42 phases, and the expression of genes in this cluster was induced by vernalization, with the highest expression in V35 followed by a rapid decline in V42, and then an increase after vernalization in V42N7. Genes in this cluster were predominantly enriched in hormone biosynthetic and metabolic process, such as indole-containing compound biosynthetic process, melatonin biosynthetic and metabolic process, GA biosynthetic process, cellular response to heat, and regulation of lipid biosynthetic process (Fig. 4a, b and c).

### Differentially expressed TFs

**Fig. 5:**
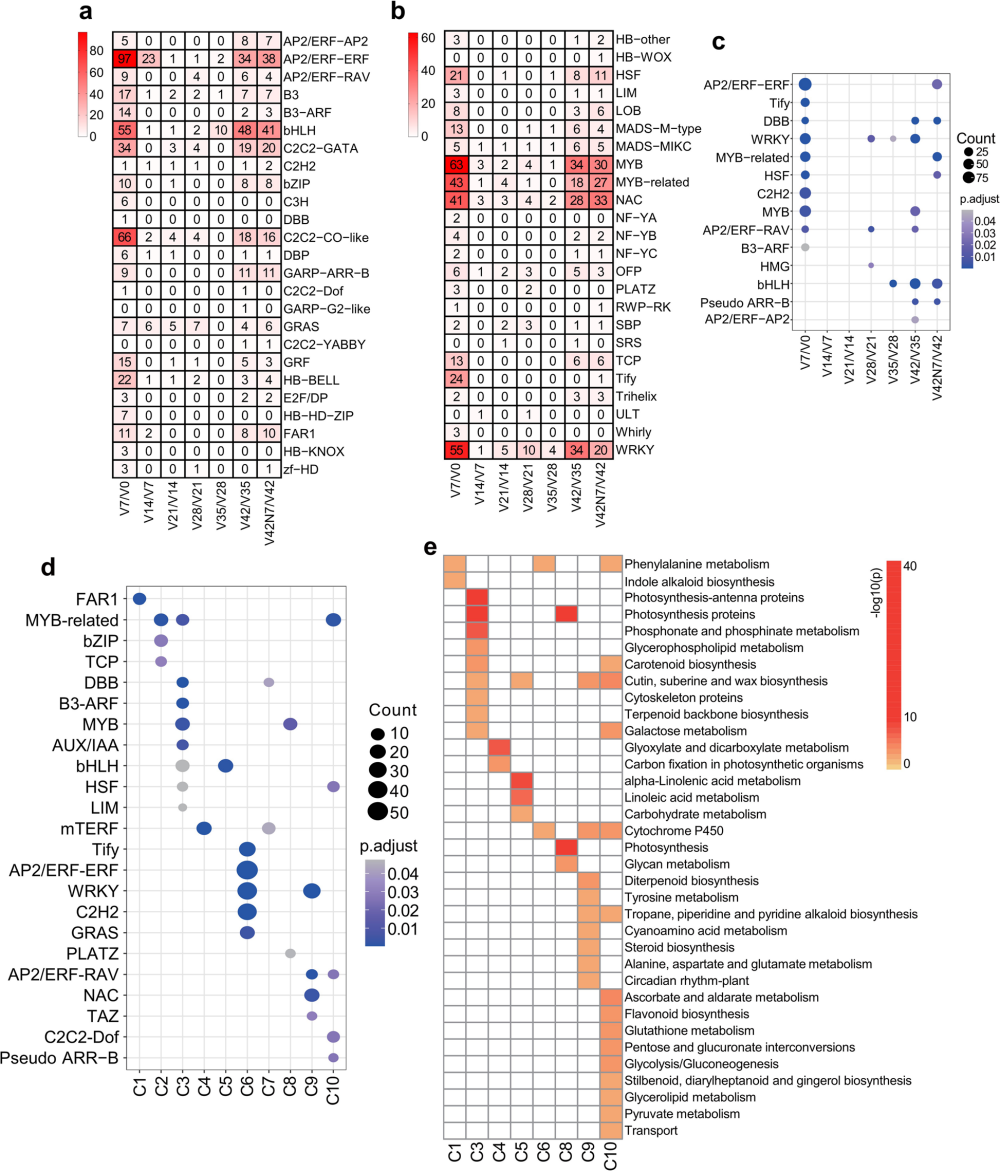
Enrichment analysis of TFs between adjacent time points and over-representation analysis of TFs in each of the 10 clusters. **a** and **b** The enriched TFs between adjacent time points (V7 vs. V0; V14 vs. V7; V21 vs. V14; V28 vs. V21; V35 vs. V28; V42 vs. V35; and V42N7 vs. V42). **c** All 14 enriched TFs between adjacent time points. The smaller the *P*-value, the higher the significance level, and the closer to the blue point; the more genes, the larger the circle. **d** Number and over-representation analysis of 23 TF gene families expressed in 10 clusters. The circle size indicates the number of TF genes in 23 TF families. The circle color represents the − log10(p) of the enrichment analysis. The TF families with *P*-value < 0.05 were considered as significantly over-represented. **e** The KEGG functional analysis of TFs enriched in eight clusters (except for C2 and C7)

Transcription factors play important roles in plant growth and development by regulating gene expression. The TFs from 49 different families were presented between adjacent time points (V7 vs. V0; V14 vs. V7; V21 vs. V14; V28 vs. V21; V35 vs. V28; V42 vs. V35; and V42N7 vs. V42) (Fig. 5a and b). Among these, *AP2/ERF-ERF* family members were the most prominent (187), followed by *bHLH* (167), *MYB* (165), *NAC* (141), *WRKY* (129), and *C2H2* (128). Various *AP2/ERF* TFs are involved in flowering regulation and stress response. The number of TF genes with peak expression levels at V7, V42, and V42N7 was more abundant at the start and end of vernalization (Fig. 5c). Ten TF families, namely *ERF* (ethylene-responsive factor), *Tify*, *DBB*, *WRKY*, *MYB-related*, *HSF* (heat shock factor), *C2H2*, *MYB*, *RAV,* and *B3-ARF,* which play important roles in plant growth regulation, had more differentially expressed members in vernalization for 1 week. Seven families were abundant in vernalization for 6 weeks, namely *DBB*, *WRKY*, *MYB*, *RAV*, *bHLH*, *Pseudo ARR-B,* and *AP2*. Six families were abundant after vernalization for 1 week, namely *ERF*, *DBB*, *MYB-related*, *HSF*, *bHLH,* and *Pseudo ARR-B*.

We further checked the TFs in these 10 different expression pattern clusters, and there were 1053, 1451, 1497, 1977, 1347, 2197, 2023, 1336, 1421, and 2068 TFs in each of the 10 clusters, respectively. Additionally, 23 TF families were found to be significantly enriched (*P* < 0.05) in these 10 different clusters using Fisher’s exact test (Fig. 5d). Cluster C3 had the most types of TFs, including *MYB*, *MYB-related*, *DBB*, *B3-ARF*, *Aux/IAA*, *bHLH*, *HSF,* and *LIM* family TFs, while Clusters C1, C3, and C4 had the lowest (one). Many TF families with significant enrichment were identified in different clusters, for example, *MYB-related* genes were enriched in clusters C2, C3, and C10, and *MYB* genes were enriched in Clusters C3 and C8. WRKY genes were enriched in Cluster 6 and Cluster 9 with high expression levels. Moreover, the *Tify*, *ERF*, *WRKY*, *C2H2,* and *GRAS* families were significantly enriched in Cluster 6. The spatiotemporal specificity of the expression of these TFs indicated their distinct roles during vernalization, and therefore they may be good candidates for further investigation.

The TFs in these clusters were analyzed via KEGG, and the results showed that the TFs in Cluster 3 were mostly enriched in photosynthesis, photosynthesis-antenna proteins, and photosynthesis proteins pathways, indicating that vernalization has a close connection with photosynthesis. In addition, TFs related to phosphonate and phosphinate metabolism, glyoxylate and dicarboxylate metabolism, and alpha-linolenic acid metabolism in KEGG pathways were significantly enriched in Clusters C3, C4, and C5, respectively (Fig. 5e).

### Expression profiles of MADS-box genes

**Fig. 6:**
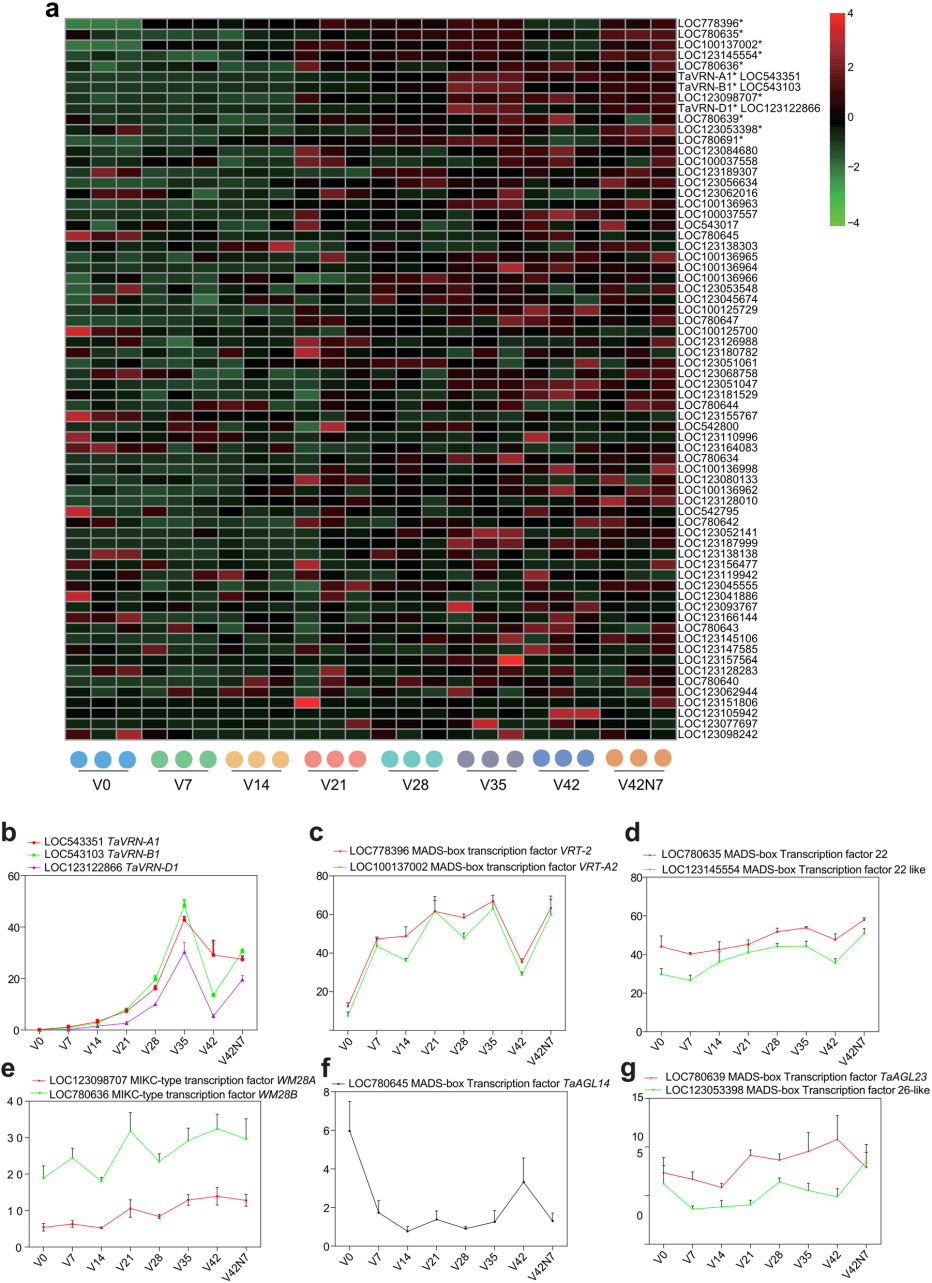
Expression analysis of MADS-box TF genes in wheat leaves before (V0), during (V7, V14, V21, V28, V35 and V42), and after (V42N7) vernalizations through RNA sequencing. **a** Heatmap analysis of MADS-box TF genes expressed in wheat leaves before, during, and after vernalization. The dot indicates one biological replicate, and gene expression is based on scaled TPM values. * Indicates that the total amount of gene expression at all time points was over 100. **b-g** The expression changes of *TaVRN-A1*, *TaVRN-B1*, and *TaVRN-D1* and another nine MADS-box genes in hexaploid wheat leaves before, during, and after vernalization based on the RNA-seq results. Error bars show the means $\pm$ SD; *n* = 3

The MADS-box TF family plays important roles in regulating plant growth and development, especially in controlling vernalization-induced flowering in cereals [33]. Here, we analyzed the expression changes of 68 MADS-box TFs before, during and after vernalization in wheat leaves based on the RNA-seq results (Fig. 6a). In wheat, *TaVRN1* (*VERNALIZATION 1*), an *APETALA1/FRUITFULL-like MADS-box* gene, has three duplications: Ta*VRN-A1* (LOC543351), *TaVRN-B1* (LOC543103), and *TaVRN-D1* (LOC123122866), and acts as a flowering activator that is induced by vernalization and aging. Here, the RNA-seq results showed that the expression levels of *TaVRN-A1*, *TaVRN-B1,* and *TaVRN-D1* were gradually induced in the leaves by vernalization from V0 to V35, reaching the highest level at vernalization for 5 weeks (V35) and then declining at vernalization for 6 weeks (V42) and increasing after vernalization (Fig. 6b). These results are similar to previous reports [11, 33, 34]. Moreover, *TaVRT-2*, which is located in Chromosome 7D and encodes a Short Vegetative Phase (SVP)-like MADS-BOX TF, has been reported to cooperate with TaVRN1 to regulate flowering induced by vernalization [34], *TaVRT-2* was rapidly induced by vernalization at V7 and showed the highest expression level at vernalization for 5 weeks, followed by a decline at vernalization for 6 weeks and an increase after vernalization for 1 week. *TaVRT-A2*, a duplicate of *TaVRT-2* in Chromosome 7A, has been reported to be involved in glume and grain elongation as well as in increasing the number of rudimentary basal spikelet in wheat. It also exhibited the same expression pattern as *TaVRT-2* (Fig. 6c). The expression of *TaVRN1* and *TaVRT2* genes in response to vernalization was consistent with previous reports [34]. Moreover, other MADS-box TFs also displayed similar expression trends under cold treatment, such as the MADS-box TFs 22 and 22-like (Fig. 6d), and MIKC-type TFs *WM28A* and *WM28B* (Fig. 6e). The *TaAGL14* gene encodes a MADS-box type II (MIKC-type) TF and forms a new subfamily of type II genes that only exist in monocotyledons along with *TaAGL15* and rice *OsMADS32* [35, 36], which also regulate floral organ identity [36]. The *TaAGL14* gene has been reported to be involved in stamen and pistil development in wheat [37] and in activating the floral switch [38]. In our results, *TaAGL14* was rapidly down-regulated by vernalization and remained at low expression levels during the V0 to V35 periods, following which it was up-regulated across the V35 to V42 periods and then down-regulated after vernalization, which indicated that it may be involved in the transition of the floral meristem and is down-regulated via the vernalization pathway (Fig. 6f).

### *ICE*-*CBF*-*COR* pathway gene expression profiles

**Fig. 7:**
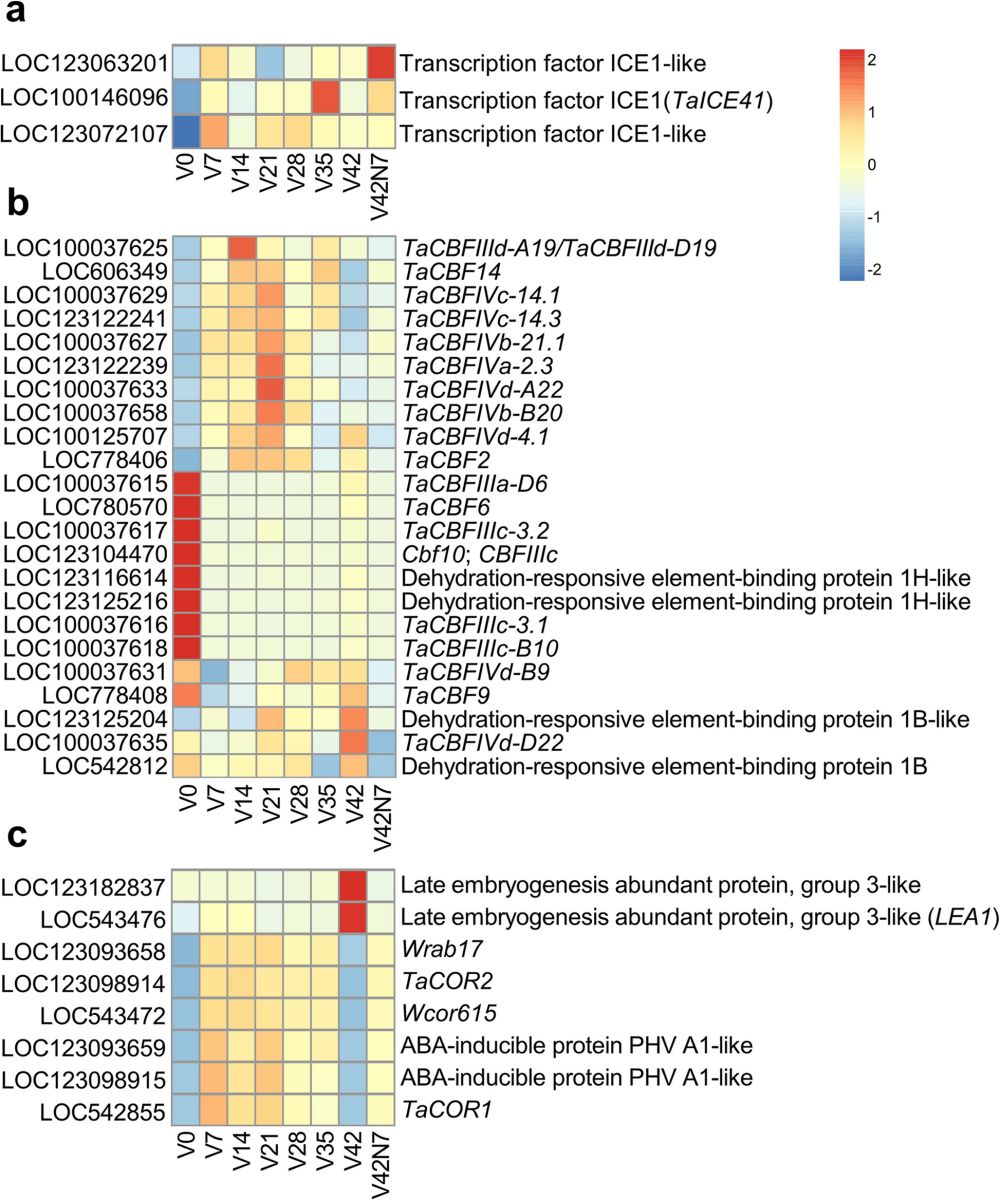
The genes identified in the *ICE*-*CBF*-*COR* pathway. Heatmaps illuminating the expression change of *ICE* genes (**a**), *CBF* genes (**b**), and *COR* genes (**c**) before (V0), during (V7, V14, V21, V28, V35 and V42), and after (V42N7) vernalizations. The gene expression is based on scaled TPM values

The *ICE*-*CBF*-*COR* pathway plays a central role in plant freezing tolerance. In this study, three *ICE* (inducer of CBF expression) genes, 23 *CBF* (C-repeat binding factor) genes, and eight *COR* (cold-responsive) genes were identified. Of these, three *ICE* genes were up-regulated during and after vernalization, and LOC100146096 (*TaICE41*) was extremely highly expressed in V35, while two TF *ICE1*-like genes LOC123063201 showed extremely high expression levels in V42N7 and LOC123072107 in V7 (Fig. 7a). Ten *CBF* genes had no expression before vernalization but were induced by vernalization and displayed high expression levels in vernalization periods, namely the *TaCBFIIId-A19/TaCBFIIId-D19*, *TaCBF14*, *TaCBFIVc-14.1*, *TaCBFIVc-14.3*, *TaCBFIVb-21.1*, *TaCBFIVd-A22*, *TaCBFIVb-B20*, *TaCBFIVa-2.3*, *TaCBFIVd-4.1,* and *TaCBF2* genes. Another eight genes were only highly expressed before vernalization in V0, namely *TaCBFIIIa-D6*, *TaCBF6*, *TaCBFIIIc-3.2*, *Cbf10/CBFIIIc*, LOC123116614, LOC123125216, *TaCBFIIIc-3.1,* and *TaCBFIIIc-B10* (Fig. 7b). Two group III late embryogenesis abundant protein genes LOC123182837 and LOC543476 were rapidly up-regulated in V42, and another six abscisic acid (ABA)-inducible protein PHV A1 genes, namely *Wrab17*, *TaCOR1*, *TaCOR2*, *Wcor615*, LOC123093659, and LOC123098915, were up-regulated by vernalization from V7 to V35, down-regulated in V42, and then up-regulated in V42N7 (Fig. 7c).

### Plant hormone-related gene changes during wheat vernalization periods

**Fig. 8:**
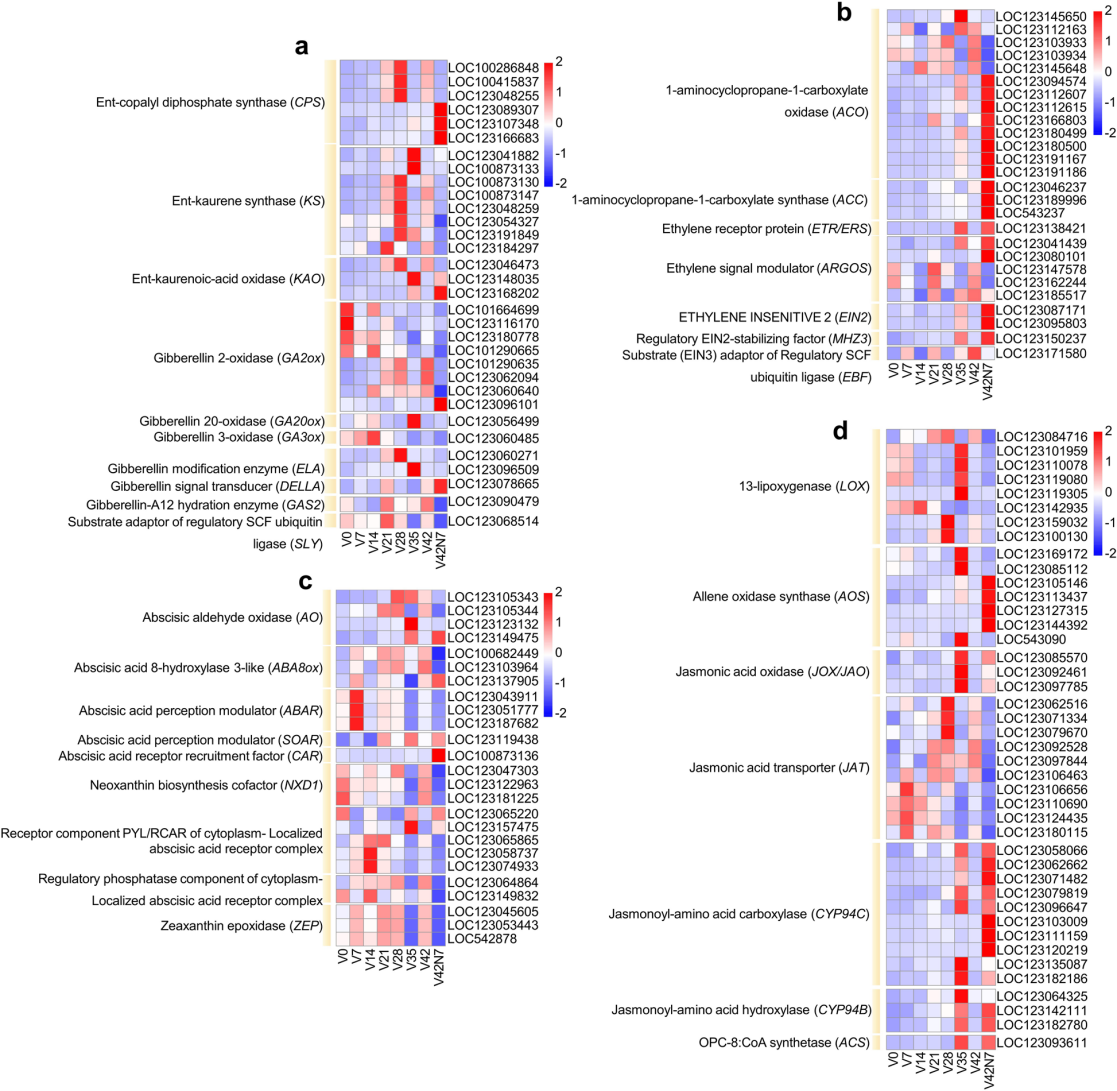
Differential expression patterns of plant hormone biosynthesis and signaling pathway genes. Heatmaps of the expression of genes related to the synthesis of gibberellin (**a**), ethylene (**b**), abscisic acid (**c**), and jasmonic acid (**d**) before (V0), during (V7, V14, V21, V28, V35 and V42), and after (V42N7) vernalizations. The gene expression is based on scaled TPM values

Genes related to plant hormone biosynthesis and signal transduction exhibited different expression levels during the vernalization phase. We analyzed the expression patterns of DEGs related to GA, ethylene, ABA, and jasmonic acid (JA). The DEGs related to phytohormone biosynthesis and signal transduction were enriched in different phases. In the GA biosynthesis pathway, we found that the ent-copalyl diphosphate synthase (*TaCPS*, six), ent-kaurene synthase (*TaKS*, eight), ent-kaurenoic-acid oxidase (*TaKAO*, three), GA 2-oxidase (*GA2ox*, eight), GA 20-oxidase (*GA20ox*, one), GA 3-oxidase (*GA3ox,* one), GA modification enzyme (*ELA*, two), and GA-A12 hydration enzyme (*GAS2*, one) genes controlled the biosynthesis of GA. In the GA signaling pathway, GA signal transducer (*DELLA*, one) and substrate adaptor of regulatory SCF ubiquitin ligase (*SLY*, one) were identified (Fig. 8a). For ethylene, 1-aminocyclopropane-1-carboxylate oxidase (*ACO*, 13) and 1-aminocyclopropane-1-carboxylate synthase (*ACC*, three) were identified for ethylene biosynthesis. In ethylene signal transduction, some genes were found, such as ethylene receptor protein (*ETR/ERS*, one), ethylene signal transducer (*EIN2*, two), ethylene signal modulator (*ARGOS*, five), regulatory EIN2-stabilizing factor (*MHZ3*, one), and substrate (*EIN3*) adaptor of regulatory SCF ubiquitin ligase (*EBF*, one) (Fig. 8b). In the ABA signaling pathway, magnesium-chelatase subunit *ChlH* (ABA receptor, three) genes were highly expressed after vernalization. Furthermore, ABA receptor recruitment factor (*CAR*, one) was specifically highly expressed at V42N7, ABA receptor *PYL4-LIke* (*PYL*, five) and protein phosphatase 2C 50-like (*PP2C*, two) genes, the key genes in the ABA signaling pathway, were found up regulated at in the ABA signaling pathway. Additionally, the homologs of *ZEP1* (zeaxanthin epoxidase, three), NEOXANTHIN-DEFICIENT 1 like (*NXD1*, three), abscisic aldehyde oxidase (*AO*, four), and ABA 8-hydroxylase 3-like (*ABA8ox*, three) genes were identified in the ABA biosynthesis pathway (Fig. 8c). For JA biosynthesis and transporting, 13-lipoxygenase (*LOX*, eight), allene oxidase synthase (*AOS*, seven), jasmonic acid oxidase (*JOX/JAO*, three), and jasmonic acid transporters (*JAT*, 10) were identified. Jasmonoyl-amino acid carboxylase (*CYP94C*, 10), jasmonoyl-amino acid hydroxylase (*CYP94B*, three), and OPC-8: CoA synthetase (*ACS*, one) were expressed highly at V35 and V42N7 and are involved in cutin, suberin, and wax biosynthesis induced by JA signaling (Fig. 8d).

## Discussion

### DEG and KEGG analysis during the vernalization period

Vernalization is critical for the flowering of winter crops and contributes to final grain yield. Here, a comprehensive transcriptome profile of vernalization was obtained via RNA-seq technology, and transcriptome analysis of different vernalization time-series treatments identified a large number of DEGs among different vernalization periods, indicating a dynamic and complex gene regulation network during vernalization.

During vernalization, the highest number of DEGs was 10,273 in V7 (5,315 down-regulated and 5,408 up-regulated) compared with V0. By contrast, V35 had the least DEGs at 467 (131 down-regulated and 336 up-regulated) compared with V28, and V42 had 6,124 DEGs (3,554 down-regulated and 2,570 up-regulated) compared with V35. There were 5,578 (3,018 down-regulated and 2,560 up-regulated) DEGs identified in V42N7 compared with V42 (Fig. 2c), which demonstrated that more molecular regulation processes occurred in the V7 period, and most of these processes might have great effects on vernalization. The GO analysis showed that DEGs related to photoprotection, photosynthesis, and response to blue light/red light/FR light/high light intensity were significantly enriched in V7/V0 and V42N7/V42 for up-regulated DEGs and in V42/V35 for down-regulated DEGs (Fig. 2e and f). This result indicated that the light-mediated signal pathway and photosynthesis were the major processes occurring in the V7 and V42N7 phases. In addition, photoperiodism, chlorophyll metabolic, pigment biosynthetic, secondary metabolite biosynthetic process, siderophore metabolic process, and also other pathways were detected during the vernalization phase. These DEGs were mainly mapped to the two largest KEGG categories of photosynthesis proteins and photosynthesis-antenna proteins (Fig. 3a and B). However, many genes were enriched in innate immune response, antifungal innate immune response, response to virus, and salt stress pathways. Vernalization (low temperature) can significantly change the membrane permeability of plant cells, which may result in decreased resistance to pathogen and omics invasion.

### TF expression profiles during vernalization

Expression analysis of TF genes showed that many TFs also participate in vernalization, such as *FAR1*, *MYB* and *MYB − related*, *DBB*, *B3-ARF*, *AUX/IAA*, *bHLH*, *mTERF*, *Tify*, *AP2/ERF − ERF*, *AP2/ERF − RAV*, *WRKY*, *C2H2*, *GRAS*, and *NAC*. Many key TFs had been identified based on their expression profiles during vernalization, and these TFs are controlling vernalization. For instance, the *APETALA2/ETHYLENE RESPONSIVE FACTOR* (*AP2/ERF*) TF family is characterized by the AP2 domain, and many *ERFs* have been reported to be involved in hormonal and abiotic stress response, such as the cold response. For instance, the overexpression of a *BpERF13* gene encoding an *AP2/ERF* family TF enhances cold tolerance [39]. This TF family is also involved in floral organ development [40] and flowering, such as rice *LATE FLOWERING SEMI* − *DWARF* (*LFS*), which encodes an AP2/ERF TF that promotes flowering under non-inductive LD conditions [41]. Our results showed that *AP2/ERF-ERF* family members were significantly enriched between V7 and V0 (97), which indicates that *ERF* may regulate the reaction to vernalization at the beginning. *FARRED IMPAIRED RESPONSE1* (*FAR1*) and *RED ELONGATED HYPOCOTYL3* (*FHY3*) are transposase-derived TFs that have also been demonstrated to play multiple roles in plant growth and development during the vegetative stage, including phytochrome A-mediated FR light signaling and circadian clock. FHY3 and FAR1 directly bind to the *CIRCADIAN CLOCK ASSOCIATED1* (*CCA1*) promoter and activate the expression of *CCA1*, *CCA1* encodes an MYB-related TF [42]. Additionally, *MYB* TFs participate in all aspects of plant development and stress responses by combining with *MYB cis*-elements in the promoters of target genes. *Circadian 1* (*CIR1*), an *MYB-related* gene, was reported to be involved in circadian regulation in *Arabidopsis*, and the constitutive expression of *CIR1* resulted in delayed flowering, longer hypocotyls, and reduced seed germination in the dark [43], FE, a phloem-specific MYB-related protein, promotes flowering through the transcriptional activation of FT and FTIP1 [44], *TaMYB72* can promote flowering in rice under long-day conditions through the up-regulation of the florigen genes *Hd3a* and *RFT1* [45]. In this study, *MYB* and *MYB-related* genes were also significantly enriched between V7 and V0 (147), V35 and V28 (80), and V42N7 and V42 (90). The *WRKY* TF gene family plays an important role in the regulation of flowering as well as in the plant cold stress response. For example, *WRKY63* acts as a dual regulator that activates *FLC* directly under non-vernalization conditions but represses *FLC* indirectly during vernalization by inducing *COOLAIR* and *COLDAIR* transcriptional activation, thus regulating vernalization-induced flowering [46], WRKY12 and WRKY13 modulate flowering time under SD conditions oppositely through interacting with FUL, and they also interact with the DELLA protein GAI and RGL1 [47]. They participate in the control of age-mediated flowering under SD conditions by physically interacting with SPLs and co-regulating the target gene *miR172b* [48]. WRKY75 directly binds to the promoter of *FT* and positively regulates flowering, and it also interacts with DELLA proteins to positively regulate flowering in *Arabidopsis* [49]. *WRKY* genes were enriched between V7 and V0 (55), V42 and V35 (34), and V42N7 and V42 (20). *bHLH* TFs have been reported to play an important role in the pathways of response to light and temperature in plants [50], and were also significantly enriched between V7 and V0 (55), V42 and V35 (48), and V42N7 and V42 (41). Here, we identified LOC100137002, an SVP-like MADS-box gene *TaVRT-A2*, which was up-regulated after vernalization and exhibited the same expression pattern as *TaVRT-2*: another SVP-like MADS-box gene that has been confirmed to interact with TaVRN1 to regulate flowering induced by vernalization.

### Genes in the* ICE*-CBF-*COR* pathway analysis during the vernalization period

Low-temperature stress profoundly influences plant growth and development, resulting in decreased crop yields. Wheat can adapt to cold conditions via cold acclimation and vernalization mechanisms. Cold acclimation allows wheat to overwinter and survive freezing temperatures, and vernalization is a necessary element that is crucial for flowering [51]. The phenomenon whereby wheat plants subjected to non-freezing temperatures increase their freezing tolerance is known as cold acclimation, which includes the expression of certain cold-induced genes that function to stabilize membranes against freeze-induced injury [52], The *ICE*-*CBF*-*COR* pathway is associated with this cold stress response [53–56]. *ICE* genes encode MYC family TFs belonging to a subfamily of bHLH, and *ICE* positively regulates *CBF* expression in the upstream region of the low-temperature signaling pathway [57–59], The key regulators of plant freezing tolerance *CBF*/DREB TFs belong to the AP2/ERF family, which induces the expression of *COR* genes and enhances freezing tolerance [54, 55, 60]. The short-term exposure to low and non-freezing temperatures is sufficient to induce cold acclimation and occurs in a variety of plant tissues [61], By contrast, vernalization is limited to the shoot apical meristem, and long-term exposure is required for vernalization [62]. The allelic variation in *VRN1* determines the differences in freezing tolerance, and *VRN1* regulates flowering in temperate cereals via the vernalization pathway [63–65]. A previous study identified *ICE*, *CBF,* and *COR* genes for cold defense in wheat [53, 56, 58, 66], In this study, three *ICE* genes showed different high expression levels in different periods. *TaICE41* was extremely highly expressed in V35, while LOC123063201 in V42N7, and LOC123072107 in V7 (Fig. 7a). It has been reported that the over-expression of *TaICE41* in transgenic *Arabidopsis* enhances freezing tolerance [46], and TaMYC2 regulates cold tolerance by physically interacting with TaICE41 and TaJAZ7, and TaJAZ7 also physically interacts with TaICE41 [47]. Ten *CBF* genes displayed high expression levels during vernalization, and eight genes were highly expressed before vernalization in V0 but exhibited relatively low expression levels during and after vernalization (Fig. 7b). Two *COR* genes (LOC123182837 and LOC543476) were rapidly up-regulated in V42, and another six *COR* genes were up-regulated during the vernalization period from V7 to V35 and were down-regulated in V42 (Fig. 7c). The genes identified herein may be used to enhance cold tolerance during wheat breeding.

### Plant hormone-related gene expression analysis during vernalization

Plant hormones regulate plant growth and development by integrating the signal response of endogenous and exogenous stimuli, and they have also been reported to regulate flowering. Herein, a series of heatmaps of plant hormone pathways during vernalization were constructed for analysis. The mechanisms of flowering regulation by phytohormones are well documented. For instance, GAs plays a key role in controlling flowering and are involved in the floral transformation [67, 68]. Mutants of GA synthesis deficient (*ga1-3* and *ga1-6*) and GA-insensitive (*gai*) in *A. thaliana* showed changed flowering times, with *ga1-3* presenting a late-flowering phenotype under long-day conditions and no flowering under short-day conditions [4]. This indicates that GA is required for initiating flowering in *Arabidopsis.* The GA biosynthesis pathway has been well defined in *Arabidopsis*, and it comprises seven key enzymes: ent-copalyl diphosphate synthase (CPS), ent-kaurene synthase (KS), ent-kaurene oxidase (KO), ent-kaurenoic acid oxidase (KAO), GA 20-oxidase, GA 3-oxidase, and GA 2-oxidase [69]. According to our results, six *TaCPS*, eight *TaKS*, eight *GA2ox*, one *GA20ox,* and one *GA3ox* genes were identified, among which three *TaCPS* were up-regulated in V21, V28, and V42 and three were up-regulated in the V42N7 phase. Five *TaKS* were significantly up-regulated in V28, as were two in V35 and one in V42 (Fig. 8a). In GA signal transduction, GID1 functions as a GA receptor, and the DELLA protein is a negative regulator thereof. The GA-GID1 complex degrades the DELLA protein to positively regulate the pathway [70, 71]. Therefore, the degradation of DELLA proteins is required to activate flowering. In this study, one DELLA was identified and up-regulated in the first, third, and sixth vernalization periods (V7, V21, and V42) and after the vernalization phase (V42N7), but it was down-regulated in the second, fourth, and fifth vernalization periods (V14, V28, and V35). This indicates that GA also features during vernalization. Jasmonate has been reported to function as an important immune signal for various plant diseases and is involved in regulating plant growth and developmental processes [72], as well as controlling flowering time in *A. thaliana* [73].

Here, JA biosynthesis-, transport-, and metabolic-related genes were identified, and three *LOX* genes (LOC123101959, LOC123110078, and LOC123119080) were down-regulated during the vernalization period from V2 to V4 but up-regulated in V35 and down-regulated during the vernalization period from V35 to V42N7. Four *AOS* genes (LOC123105146, LOC123113437, LOC123127315, and LOC123144392) were up-regulated in V42N7, and three *AOS* genes (LOC123085112, LOC123100130, and LOC543090) were up-regulated in V35. Three *JOX/JAO* genes were up-regulated in V35, down-regulated in V42, and up-regulated in V42N7, and *JAT* genes were also up-regulated during vernalization. Six *CYP94C* genes showed induced expression in V35 and V42N7, three in V42N7, and one in V35. Two *CYP94B* genes exhibited induced expression in V35 and V42N7, and one in V35 was highly expressed. The *ACS* gene showed high expression at V35 and V42N7 and is involved in cutin, suberin, and wax biosynthesis induced by JA signaling (Fig. 8d). The result indicated that JA may regulate vernalization in wheat.

Ethylene is a gaseous phytohormone that acts as a plant flowering regulator. Ethylene biosynthesis starts from S-adenosyl-L-methionine (SAM) via two exclusive enzymatic reactions and is catalyzed by 1-aminocyclopropane-1carboxylic acid (ACC) synthase (ACS) and ACC oxidase (ACO) [74]. Our results showed that *ACS* genes were mainly expressed in V35 and V42N7, and *ACO* genes were specifically expressed in V42N7. In the ethylene signal transduction pathway, ethylene is perceived by the receptors ETR1 (ethylene resistant 1) and EIN2 (ethylene insensitive 2) and plays a central role in ethylene transduction [75], OsMHZ3 interacts with OsEIN2 as a stabilizer of OsEIN2 [76]. In *A. thaliana*, *ETHYLENE INSENSITIVE3* (*EIN3*) and *EIN3*-like (*EIL*) TFs act downstream of EIN2, and EIN3 BINDING F-BOX1 (EBF1) and EBF2 function in the ethylene perception process by regulating EIN3/EIL turnover [77, 78]. The *ETR/ERS* (LOC123138421), *EIN2* (LOC123087171, LOC1230958030), and *MHZ3* (LOC123150237) genes were identified in V35 and V42N7, and the *EBF* (LOC123171580) gene was up-regulated in the V7, V21, V35, and V42 periods (Fig. 8b), which suggests that ethylene may participate in the control of vernalization.

Abscisic acid is a carotenoid-derived phytohormone that plays vital roles in the response to biotic and abiotic stresses as well as in various physiological and developmental processes [79–81]. Additionally, ABA has negative effects on the floral transition in *A thaliana* [82–84]. In the ABA biosynthetic pathway, zeaxanthin epoxidase (ZEP), 9-cisepoxycarotenoid dioxygenase (NCED), and aldehyde oxidase (AO) are key enzymes, and ZEP catalyzes two-step reactions from zeaxanthin to epoxidation and forms all-trans-violaxanthin, while NCED catalyzes the oxidative cleavage of a 9-cisisomer of epoxycarotenoid such as 9-*cis*-violaxanthin and 9′-*cis*-neoxanthin to form xanthoxin, and AO catalyzes the oxidation of ABAld (abscisic aldehyde) [85]. ABA 8-hydroxylase catalyzes the first step of the predominant ABA catabolic pathway [86]. Three *ZEP1* genes were identified and showed high expression levels in V7, V21, V28, and V42, and the *AO* and *ABA8ox* genes showed induced expression during different vernalization periods (Fig. 8c). In the ABA signaling pathway, ABA is recognized by the receptors PYR/PYLs/RCARS/ABAR/CHLH [87–89], which in turn bind to and inhibit the enzymatic activity of clade A PP2Cs [88]. When ABA is insufficient, PP2Cs interact with and inactivate SNF1-related kinase2 (SnRK2) via dephosphorylation [90], and in the presence of ABA, PP2Cs are inhibited and SnRK2 are free to phosphorylate ABRE BINDING FACTORs/ABRE-BINDING PROTEINs (ABFs/AREBs) TFs, thereby activating ABA-responsive genes [91]. Three ABA receptors of *ABAR/CHLH* genes (LOC123043911, LOC123051777, LOC123187682) were identified and strongly induced after vernalization, with the *PYL/RCAR* and *PP2C* genes showing differential accumulation during different vernalization periods (Fig. 8c). Therefore, the ABA biosynthesis and signaling pathways participate in regulating the transformation process induced by vernalization.

## Conclusions

Vernalization, as an important character of winter crops, promotes plant flowering and is directly linked to final crop production. Thus, it is necessary to elucidate the regulatory mechanisms involved in vernalization. Therefore, transcriptome analysis of wheat leaves over various vernalization periods was conducted herein, and many key genes with specific expression patterns and genes in the *ICE*-*CBF*-*COR* pathway were identified, implying their considerable influence on the vernalization process. According to our results, photoperiod, photoprotection, and photosynthesis pathways play important roles in regulating wheat flowering during vernalization. This work also elucidated the potential regulatory mechanisms of phytohormone metabolism and TF expression during vernalization, which could provide numerous candidate genes for further functional characterization. These findings are a valuable resource for future investigations on the molecular mechanisms of wheat vernalization.

### Supplementary Information

**Additional file 1: Table S1.** DEGs identified in all 7 comparisons between adjacent time points.

**Additional file 2: Table S2.** GO enrichment analysis of up/down-regulated DEGs between adjacent time points according to the biological process.

**Additional file 3: Table S3.** KEGG analysis of up-regulated DEGs between adjacent time points.

**Additional file 4: Table S4.** KEGG analysis of down-regulated DEGs between adjacent time points.

**Additional file 5: Table S5.** The most GO enrichment analysis of up/down-regulated DEGs in 10 clusters according to the biological process.

**Additional file 6: Table S6.** The expression analysis of identified MADS-box genes before, during and after vernalization.

**Additional file 7: Table S7.** Differential expression patterns of plant hormone biosynthesis and signaling pathway related genes.

## Data Availability

All data generated or analyzed during this study were included in this published article and the additional files. The datasets generated and analyzed during the current study are available in National Center for Biotechnology Information (NCBI) BioProject database under accession number PRJNA963171.

## Abbreviations

GO: Gene Ontology
KEGG: Kyoto Encyclopedia of Genes and Genomes
SCC: Spearman’s Correlation Coefficient
PCA: Principal Component Analysis
AP2/ERF: APETALA2/ETHYLENE RESPONSIVE FACTOR
RAV: Related to ABI3/VP1
B3: B3 DNA-binding domain
ARF: Auxin Response Factors
bHLH: basic Helix-loop-helix
C2C2-TAGA: Zinc finger TAGA transcription factor
C2H2: Cys2His2 (C2H2) zinc-finger transcription factor
C3H: Zinc finger CCCH domain-containing protein
bZip: Basic region leucine zipper
HB-HD-ZIP: Homeodomain-Leucine Zipper
HSF: Heat Shock Factor
NAC: NAM, ATAF1/2, CUC1/2
WRKY: WRKYGOK
FAR1: FAR-RED IMPAIRED RESPONSE 1
SRS: SHI RELATED SEQUENCE
NF-YA: Subunit A of nuclear factor-Y
NF-YB: Subunit B of nuclear factor-Y
NF-YC: Subunit C of nuclear factor-Y
GARP: Golden2, ARR-B, Psr1 family
GRAS: GAI, RGA and SCR family
GRF: Growth-regulating factor
DBP: D-BOX-BINDING PAR bZIP transcription factor
DBB: Double B-Box
dof: DNA binding with one finger
HB-KNOX: Homeobox-KNOTTED-LIKE HOMEOBOX
ZF-HD: Zinc Finger Homeodomain
OFP: Ovate Family Proteins
PLATZ: Plant AT-rich sequence and zinc-binding protein
SBP: Squamosa promoter binding protein
TCP: TEOSINTE BRANCHED1/CYCLOIDEA/PROLIFERATING CELL FACTOR
mTERF: Mitochondrial Transcription Termination Factor
Aux/IAAs: AUXIN/INDOLE ACETIC ACIDs
ICE: Inducer of CBF expression
CBF: C-repeat binding factors
COR: Cold responsive genes
